# *In vivo* evaluation of the potential protective effects of prolactin against damage caused by methylmercury

**DOI:** 10.1590/1414-431X2022e11976

**Published:** 2022-07-13

**Authors:** L. Cunha, L. Bonfim, G. Lima, R. Silva, L. Silva, P. Lima, V. Oliveira-Bahia, J. Freitas, R. Burbano, C. Rocha

**Affiliations:** 1 Laboratório de Citogenética Humana, Universidade Federal do Pará, Belém, PA, Brasil; 2 Laboratório Multidisciplinar de Morfofisiologia Animal, Universidade Federal do Pará, Belém, PA, Brasil; 3 Laboratório de Morfofisiologia Aplicada à Saúde, Universidade do Estado do Pará, Belém, PA, Brasil; 4 Laboratório de Citogenética, Universidade do Estado do Pará, Belém, PA, Brasil; 5 Laboratório de Biologia Molecular, Hospital Ophir Loyola, Belém, PA, Brasil; 6 Instituto Federal de Educação, Ciência e Tecnologia do Pará, Diretoria de Pós-Graduação, Pesquisa e Inovação (DPI), Belém, PA, Brasil

**Keywords:** Mercury, Prolactin, Histological evaluation, Biochemical parameters, Genotoxicity

## Abstract

Non-biodegradable metals such as mercury accumulate in living organisms during life (bioaccumulation) and also within trophic webs (biomagnification) and may reach high concentrations in humans. The contamination of humans by mercury in drinking water and food may be common, in particular in riverside communities that have a diet rich in fish. *In vitro* studies of human cell lines exposed to the cytotoxic and mutagenic effects of methylmercury have shown that prolactin has potential cytoprotective properties and may act as a co-mitogenic factor and inhibitor of apoptosis. The present *in vivo* study investigated the protective potential of prolactin against the toxic effects of methylmercury in the mammal *Mus musculus*. Histological and biochemical analyses, together with biomarker of genotoxicity, were used to verify the protective potential of prolactin in mice exposed to methylmercury. The reduction in kidney and liver tissue damage was not significant. However, results of biochemical and genotoxic analyses were excellent. After prolactin treatment, a significant reduction was observed in biochemical parameters and mutagenic effects of methylmercury. The study results therefore indicated that prolactin has protective effects against the toxicity of methylmercury and allowed us to suggest the continuation of research to propose prolactin in the future, as an alternative to prevent the damage caused by mercury, especially in populations that are more exposed.

## Introduction

The principal source of human exposure to heavy metals is the consumption of contaminated food, in particular fish, which contains toxic elements such as arsenic, cadmium, lead, chromium, and mercury (1). Mercury is one of the most common and deleterious organ-specific contaminants in the world (2).

In general, mercury has adverse effects on the polymerization of tubulin, which promotes the contraction of the chromosomes in the metaphase and the delay in anaphasic movement and centromeric division (3) and may also cause chromosomal anomalies such as polyploidy (2). An additional effect is the production of free radicals, which may permanently damage the DNA (4).

Since 1993, the International Agency for Research on Cancer (IARC) has classified methylmercury (MeHg) in group 2B, that is, potentially carcinogenic to humans. As they are not biodegradable, metals such as mercury accumulate in living organisms during life (bioaccumulation) and also within trophic webs (biomagnification or trophic magnification) and can reach high concentrations in humans (5,6).

The transfer of mercury to aquatic systems from mining operations and through the lixiviation of soils following deforestation is considered to be the principal source of contamination of the Amazon basin (7). In the Brazilian Amazon, studies have found high levels of MeHg in a number of carnivorous fish species (8). Epidemiological studies have found evidence of neurological deficits in the populations of fishing communities that depend on fish for their survival (9). A diet rich in fish is the principal source of human exposure to this type of mercury (10).

Prolactin is a protein-based hormone of the same family as the growth hormone and placental lactogens, and it is produced and secreted principally by the lactotrophs of the adenohypophysis. In addition to the hypophysis, prolactin is known to be synthesized and secreted by the brain, placenta, uterus, mammary gland, immunocompetent cells, lymphoid cells of the bone marrow, and sweat glands. In addition to stimulating the production of milk by the mammary glands (lactogenesis), prolactin also supports mammogenesis and galactopoiesis, and has further 300 biological functions, being involved in homeostasis, immune regulation, osmotic balance, and angiogenesis, as well as affecting cell growth and proliferation, and acting as a neurotransmitter (11,12).

In all vertebrate classes, prolactin is involved in the osmotic balance (of water and electrolytes). In mammals, prolactin receptors are present in kidney cells and other organs involved in this process. Many of the effects of prolactin are associated with cell proliferation. In the skin, it stimulates the proliferation of melanocytes and keratinocytes, and may influence the growth of the hepatocytes, inducing a number of genes related to the growth of liver cells. The development of the intestinal mucosa and vascular smooth muscle, the proliferation of β cells in the pancreas, astrocytes, and other cells of the immune system have all been associated with prolactin (11).

Prolactin activates the transduction signal pathways by activating its receptor. The prolactin receptor is associated constitutively with Janus kinase 2 (JAK2) proteins. When activated, JAK2 phosphorylates the tyrosine residues in target proteins, including its own receptor, and the proteins Stat 1, Stat 2, and, primarily, Stat 5. These proteins dimerize and translocate themselves until the signal reaches the nucleus, activating the gene promotors that are responsive to prolactin. In addition to the JAK/Stat pathway, other pathways may be activated by prolactin receptors, such as the Ras/Raf/MAP kinase pathway, which may be related to the proliferative effects of this hormone (11,13).

All these functions of prolactin and the signaling pathways it activates may be related to its protective effects in different types of tissue and experimental situations. The protective potential of prolactin against the effects of MeHg have been observed *in vitro*, where the hormone attenuated the impacts of the compound on cell viability, the response of the immune system cells, and the mercury-induced cytotoxicity and mutagenicity (13,14).

Based on these observations, the present *in vivo* study investigated the protective potential of prolactin against the toxic effects of MeHg in mice.

## Material and Methods

Healthy young adult mice (*Mus musculus*) of both sexes were obtained from the vivarium of the Evandro Chagas Institute, in Belém, and kept in the vivarium at Pará State University (UEPA) with five animals per cage. The males and females were kept separately in an environment with a controlled temperature of 22±3°C and a 12-h light/dark cycle, and free access to water and feed (commercial food) (15).

### Treatments

The animals were divided into six groups each containing 10 animals, including five of each sex. Once acclimatized, the animals were exposed to the treatments with CH_3_HgCl (Sigma-Aldrich^®^, USA) and prolactin (Sigma-Aldrich^®^), both of which were diluted in distilled water (mother solution) or 0.9% saline solution (working solution) for 45 days. These concentrations were defined based on published studies (4,16). The animals were given the MeHg by gavage and the prolactin by subcutaneous injection. The treatments were as follows: control group (CN: subcutaneous injection of 0.9% saline solution); MeHg group (30 μg/kg CH_3_HgCl per day); PRL 25 group (25 μg/kg prolactin every 12 h); PRL 250 group (250 μg/kg prolactin every 12 h); MeHg+PRL 25 group (30 μg/kg CH_3_HgCl per day and 25 μg/kg prolactin every 12 h), and the MeHg+PRL 250 group (30 μg/kg CH_3_HgCl per day and 250 μg/kg of prolactin every 12 h).

On each day, before the application of the first treatment of the day, the animals were weighed and the solutions for each treatment were prepared based on the mean body mass of each cage. At the end of each treatment period, the animals were anesthetized for cardiac puncture and then euthanized (whenever necessary) by cervical lesion, prior to the extraction of the organs/tissues for the bioassays.

The study followed the guidelines of the Brazilian legislation for the breeding and use of animals in experiments (federal law 11,794 of 2008) and the Ethical Guidelines of the Brazilian College of Animal Experimentation (COBEA), and was conducted in accordance with Brazilian guidelines for the care and use of animals on scientific research and teaching - DBCA (17). The study was approved by the UEPA Committee on Ethics for the Use of Animals in Research (CEUA/UEPA) under protocol number 16/2017.

### Biochemical analyses

Blood samples were analyzed to determine the serum levels of urea, creatinine, aspartate aminotransferase (AST), alanine aminotransferase (ALT), mercury, and prolactin using a commercial kit (Labtest, Brazil). The total mercury was determined by the procedure described by Yasutake et al. (18), in which the blood was first hemolyzed using distilled water (1:50) and the concentrations of mercury in the homogenates (100 μL) and hemolyzed blood (100 μL) were determined by the oxygen combustion-gold amalgam method using an MD-A atomic mercury absorption analyzer (https://mercuryanalyser.com/index.html). The plasmatic prolactin levels were measured by ELISA (Mouse Prolactin DuoSet; R&D Systems, Brazil) using the DuoSet ELISA development kit, which contains all the basic components necessary for the development of ELISAs for the measurement of the natural and recombinant mouse prolactin.

### Histological analysis

The histological analysis was carried out as previously described by Prophet et al. (19) and Alnoaimi et al. (20). The organs extracted for the histological analysis (kidneys and liver) were fixed in 10% formaldehyde before being processed for analysis. The samples were first dehydrated in an increasing ethanol series (70, 80, 90, 95, and 100%), immersed in xylol for diaphanization (clarification), and embedded in paraffin blocks. Sections (5-μm thick) were then stained with hematoxylin and eosin for analysis under a light microscope at a magnification of 400×. Considering the number of quadrants observed in each slide, the frequency of the cellular alterations in each animal was classified as: (0) not detected, (0+) rare, (+) low, (++) moderate, and (+++) high. To calculate the Degree of Tissue Change (DTC), the histopathological changes in each organ were classified into three phases of damage; phase I (FI) refers to reversible cytological and tissue damage that does not affect organ functionality, phase II (FII) indicates cytological and tissue damage that has moderate reversibility and does not alter the organ's functionality, and phase III (FIII) refers to irreversible cytological and tissue damage that leads to organ dysfunction. DTC was quantified using the formula: DTC = (1 × ΣFI) + (10 × ΣFII) + (100 × ΣFIII). ΣFI, ΣFII, and ΣFIII are calculated by the total number of histopathological damages observed in each phase.

### Micronucleus test

Following euthanasia, the leg of each animal was dissected for the removal of the femur, whose extremities were cut off to expose the bone marrow. Bovine fetal serum (BFS) was injected into the bone cavity using a 1-mL syringe, which caused the marrow to exit into an individually-labeled test tube. This material was centrifuged at 71.5 *g* for 5 min at room temperature, the supernatant was discarded, and the sediment was resuspended in 0.5 mL of BFS. Two or three drops of this suspension were then dripped onto a clean slide, spread, and air-dried at room temperature. Once dried and fixed in absolute ethanol, the slide was stained with Leishman to differentiate the polychromatic erythrocytes (PCE), normochromatic erythrocytes (NCE), and micronucleated polychromatic erythrocytes (MNPCEs) (15,21).

The slides were examined for micronuclei under a microscope at a magnification of 1000×, with 1,000 erythrocytes being examined per slide (2,000 per animal) in a blind manner. The significance of the variation in the frequency of abnormalities among the experimental groups was evaluated using a one-way analysis of variance (ANOVA) followed by Tukey's *post hoc* test, with a significance level of 5%. In addition to the micronucleus test, the cytotoxicity of the substances tested in the bone marrow was analyzed by the percentage of PCE in total erythrocytes. The reduction in this percentage indicates excessive toxicity in the bone marrow of the animals (21).

## Results

**Figure 1:**
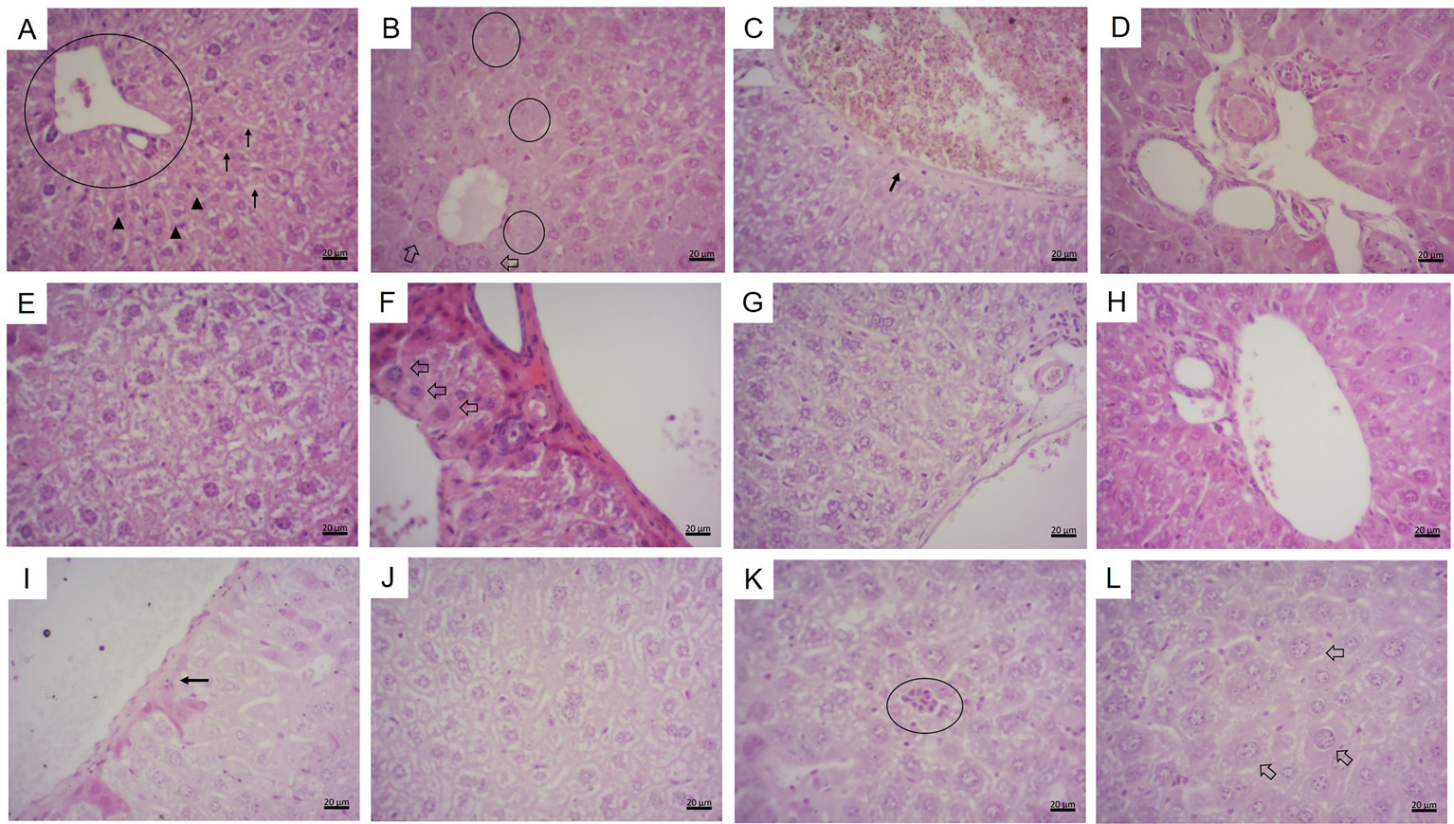
H&E liver histology. **A**, Female - negative control group: normal liver tissue, portal space (circle), sinusoids (arrows), and hepatocytes (arrowheads). **B**, Male - MeHg group: hypertrophied hepatocytes (arrows) and areas with cells in karyolysis (circles). **C,** Male - MeHg group: triad vein wall thickening (arrow). **D**, Male - MeHg group: triad with an increase in the number of structures. **E**, Male - PRL 25 group: area with edema. **F**, Female - PRL 25 group: hypertrophied hepatocytes (arrows). **G**, Female - PRL 250 group: area with edema. **H**, Male - PRL 250 group: normal liver tissue. **I**, Male - MeHg+PRL 25 group: triad vein wall thickening (arrow). **J**, Female - MeHg+PRL 25 group: area with edema. **K**, Male - MeHg+PRL 250 group: inflammatory infiltrate (circle). **L**, Female - MeHg+PRL 250 group: hypertrophied hepatocytes (arrows). Scale bar: 20 μm. MeHg: methylmercury; PRL 25 and PRL 250: 25 and 250 μg/kg prolactin, respectively, every 12 h.

**Figure 2:**
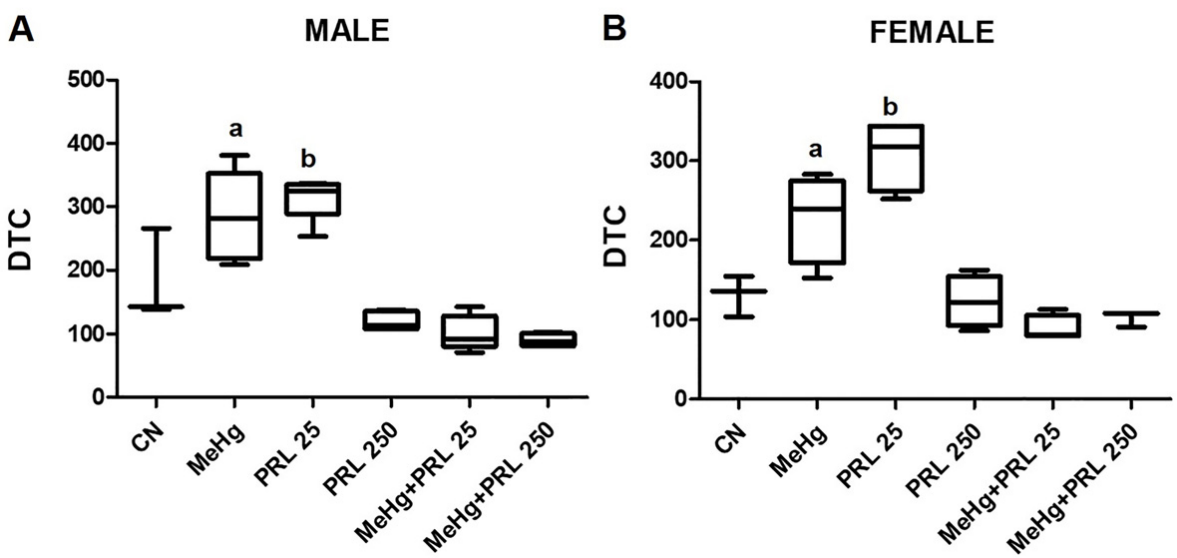
Degree of tissue change (DTC) in the liver in each treatment group for (**A**) males and (**B**) females. Data are reported as medians and interquartile range. ^a^P<0.01 compared to all other treatment groups (except the PRL 25 group); ^b^P<0.01 compared to all other treatment groups (except the MeHg group) (ANOVA with Tukey's post-test). CN: Control group; MeHg: methylmercury; PRL 25 and PRL 250: 25 and 250 μg/kg prolactin, respectively, every 12 h.

In general, the histological sections of the mouse liver presented hepatocytes with well-defined limits, granulated cytoplasm, central spherical nuclei, and whole sinusoids, as well as a clear view of the centrilobular vein and the *porta hepatis*. Congested blood vessels, areas of hypertrophied hepatocytes, and cells with pyknotic nuclei were observed in the liver sections of mice of all the experimental groups, including the control, of both sexes, and at higher or lower frequencies (Table 1 and Figures 1 and 2).

**Table 1:** Alterations observed in the liver tissue of the mice of each treatment group.

	Females						Males					
	CN	MeHg	PRL 25	PRL 250	MeHg/PRL 25	MeHg/PRL 250	CN	MeHg	PRL 25	PRL 250	MeHg/PRL 25	MeHg/PRL 250
Hypertrophied hepatocytes	0+	0+	+	0+	0+	0+	0+	+	+	++	0+	0+
Inflammatory infiltration	0	+	++	++	0+	0+	0+	+	+	+	0+	0+
Pyknosis	+++	+	+++	+	0+	++	+++	++	+++	+	+	+
Edema	0	0+	++	+++	+++	+	+	+++	+++	+++	+++	++
Karyolysis	0	0+	0	0	0	0	0+	0+	0+	0	0	0
Fibrosis associated with the vessels of the triad	0	0	0	0+	0	0	0	0	0	0	0	0
Vacuolization near the vessels of the triad	0	0	0	0+	0+	0	0	0	0	0	0+	0
Thickening of the vessels of the triad	0	++	++	++	0+	+	0	++	+	+	0+	+
Increase in the number of vessels surrounding the triad	0	0+	0+	0	0	0	0	0+	0+	0	0	0
Deformed triad	0	0+	0+	0	0	0	0	0+	0+	0	0	0

(0) not detected; (0+) rare; (+) low frequency; (++) moderate frequency; (+++) high frequency. CN: control group; MeHg: methylmercury; PRL 25 and PRL 250: 25 and 250 μg/kg prolactin, respectively, every 12 h.

Both MeHg and prolactin provoked alterations in liver tissue. However, the combined treatment (MeHg+PRL) showed a tendency to decrease the frequency of some of these alterations, such as hypertrophied hepatocytes, presence of inflammatory infiltrates, and alterations in or around the *porta hepatis*. An increase in the number of vessels in the vicinity of the *porta hepatis* and the deformation of the triad were rare, being observed only in the MeHg and PRL 25 groups. In the general analysis of liver damage, one result can be considered surprising: the greatest degree of liver tissue alteration occurred in the PRL 25 groups. The protective effect of prolactin was observed in relation to lesions with areas of anucleated cells, typical of advanced karyolysis, being more evident in females.

**Figure 3:**
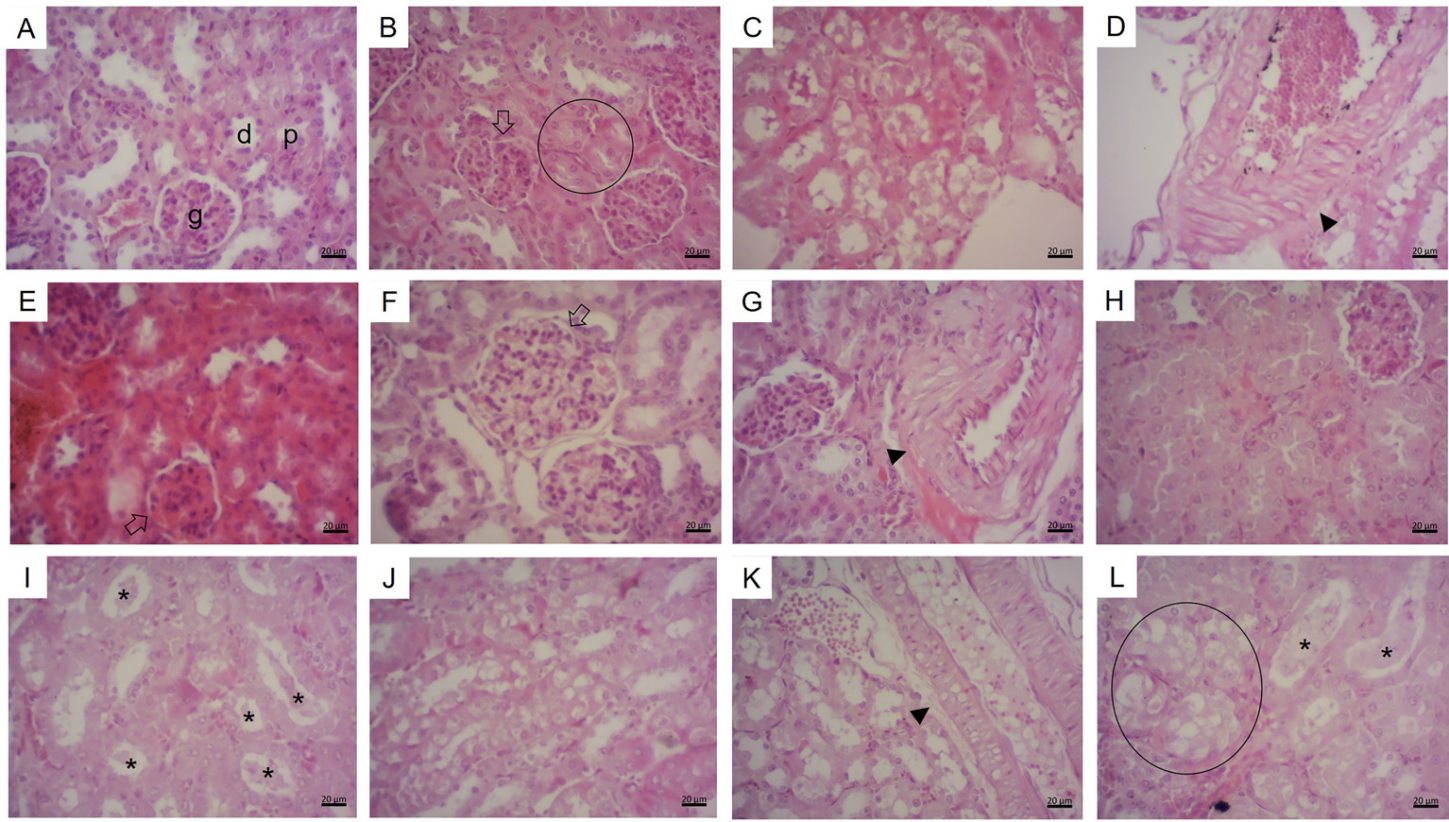
H&E kidney histology. **A**, Female - negative control group: normal renal parenchyma, glomerulus (g), proximal tubule (p), and distal tubule (d). **B**, Female - MeHg group: hypertrophied glomerulus (arrow), swollen tubules (circle). **C**, Male - MeHg group: area with cells in vacuolar degeneration. **D**, Male - MeHg group: vessel with hyaline degeneration in the media layer (arrowhead). **E**, Male - PRL 25 group: hypertrophied glomerulus (arrow). **F**, Female - PRL 25 group: hypertrophied glomerulus (arrow). **G**, Female - PRL 250 group: vessel with hyaline degeneration in the media layer (arrowhead). **H**, Male - PRL 250 group: swollen tubules. **I**, Male - MeHg + PRL 25 group: desquamation of necrotic cells in the tubules (asterisks). **J**, Male - MeHg + PRL 25 group: area with vacuolar degeneration cells. **K**, Female - MeHg + PRL 250 group: vessel with hyaline degeneration in the media layer (arrowhead). **L**, Male - MeHg + PRL 250 group: peeling of necrotic cells in the tubules (asterisks), area with cells in vacuolar degeneration (circle). Scale bar: 20 μm. MeHg: methylmercury; PRL 25 and PRL 250: 25 and 250 μg/kg prolactin, respectively, every 12 h.

**Figure 4:**
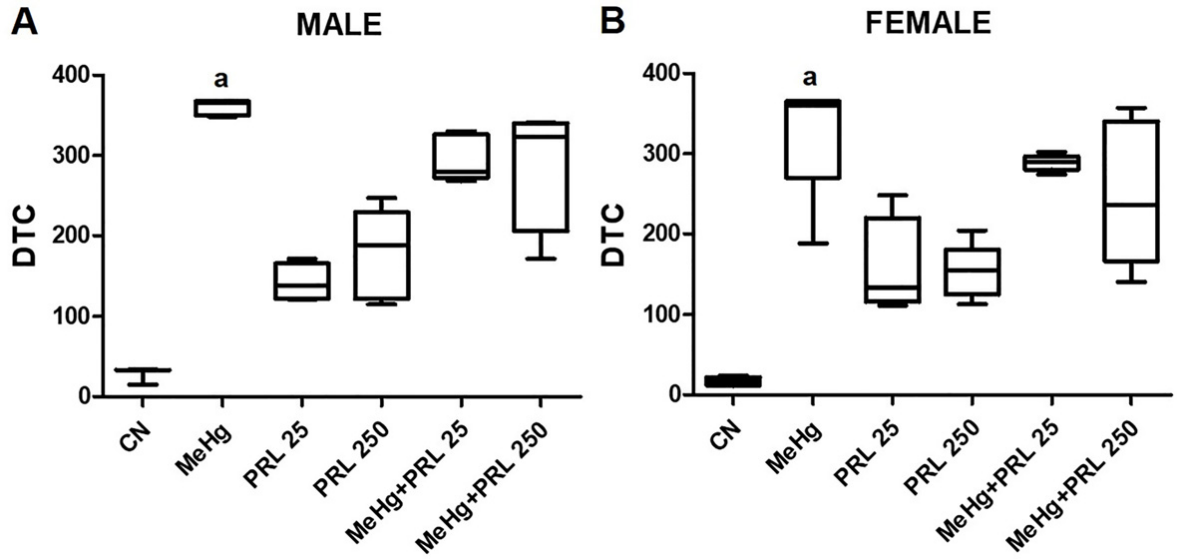
Degree of tissue change (DTC) in the kidney in each treatment group for (**A**) males and (**B**) females. Data are reported as medians and interquartile range. ^a^P<0.01 compared to the CN, PRL 25, and PRL 250 groups (ANOVA with Tukey's post-test). CN: Control group; MeHg: methylmercury; PRL 25 and PRL 250: 25 and 250 μg/kg prolactin, respectively, every 12 h.

The histological analysis of the mouse kidneys revealed tubules dispersed throughout the parenchyma and rounded glomeruli, with whole Bowman's capsules. Some alterations were observed in all the treatment groups, at different frequencies (Table 2 and Figures 3 and 4). Hypertrophied glomeruli were observed in all the treatment groups, slightly more often in females. A moderate frequency of alterations was recorded in the control and PRL (25 and 250) groups, with most quadrants containing only 1-3 altered glomeruli. In the MeHg and MeHg+PRL groups, however, more than 10 hypertrophied glomeruli were observed in most of the quadrants analyzed, reaching 30 altered glomeruli in a single quadrant. Regarding this alteration, there was no evidence of a protective action of prolactin against the effects of MeHg.

**Table 2:** Alterations observed in the kidney tissue of the mice of each treatment group.

	Females						Males					
	CN	MeHg	PRL 25	PRL 250	MeHg/PRL 25	MeHg/PRL 250	CN	MeHg	PRL 25	PRL 250	MeHg/PRL 25	MeHg/PRL 250
Hypertrophied glomeruli	++	+++	++	+++	+++	+++	0+	+++	++	++	++	++
Swollen tubules	+++	+++	0+	0+	++	+++	+++	+++	0	++	+++	+++
Peeling of the tubules	+	++	0	0	+	+	++	++	0	+	+	+++
Vacuolar degeneration	0	++	0+	0+	+++	++	0	+++	0	++	+++	++
Hyaline degeneration of the vessels	0	+++	+++	+++	+++	+++	0+	+++	+++	+++	+++	+++

(0) not detected; (0+) rare; (+) low frequency; (++) moderate frequency; (+++) high frequency. CN: control group; MeHg: methylmercury; PRL 25 and PRL 250: 25 and 250 μg/kg prolactin, respectively, every 12 h.

Vacuolar degeneration was not observed in the control group in either sex. In both males and females, this alteration was more frequent in the MeHg group than in the PRL group. In males, a lower frequency of this alteration was observed in the MeHg+PRL 250 group than in the MeHg group. Another alteration observed very frequently in all treatment groups, with the exception of the control, was the hyaline degeneration of blood vessel walls.

**Figure 5:**
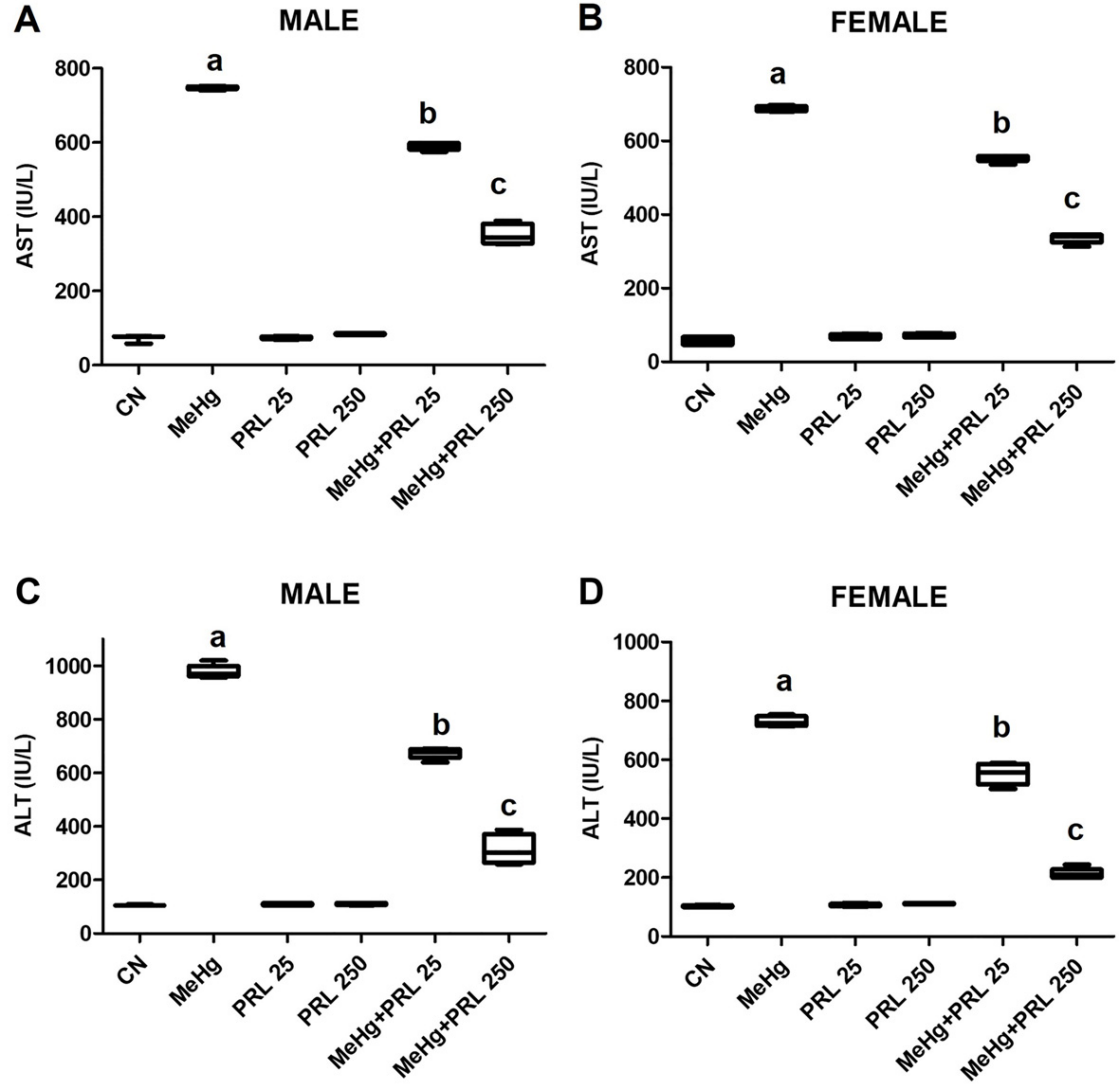
Hepatic markers in males (**A** and **C**) and females (**B** and **D**) of each treatment group. Data are reported as medians and interquartile range. ^a,b,c^P<0.01 compared to all other treatments (ANOVA with Tukey's post-test). CN: control group; MeHg: methylmercury; PRL 25 and PRL 250: 25 and 250 μg/kg prolactin, respectively, every 12 h.

**Figure 6:**
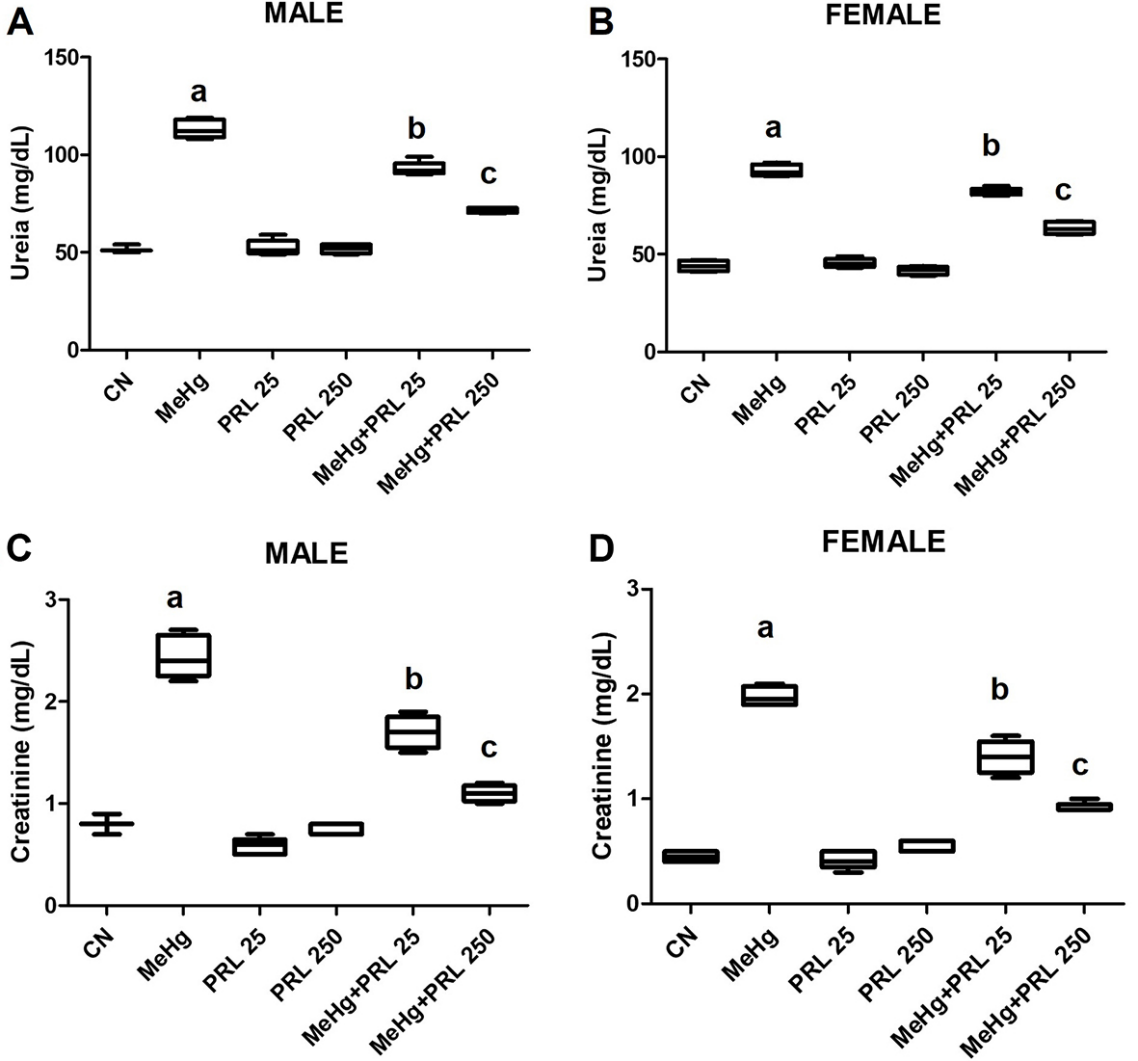
Renal markers in males (**A** and **C**) and females (**B** and **D**) of each treatment group. Data are reported as medians and interquartile range. ^a,b,c^P<0.01 compared to all other treatments (ANOVA with Tukey's post-test). CN: control group; MeHg: methylmercury; PRL 25 and PRL 250: 25 and 250 μg/kg prolactin, respectively, every 12 h.

**Figure 7:**
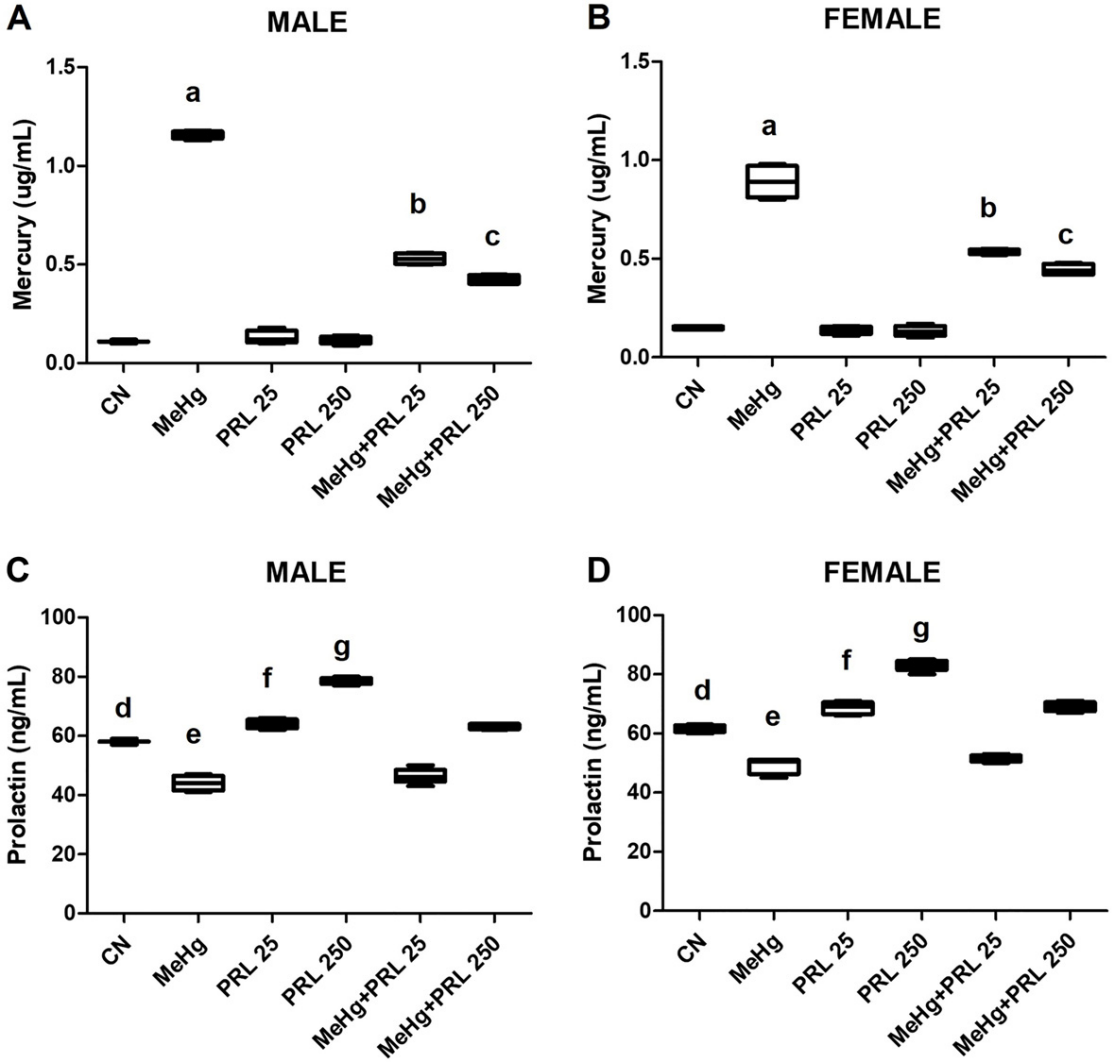
Serum mercury level (**A** and **B**) and serum prolactin level (**C** and **D**) of each treatment group. Data are reported as medians and interquartile range. ^a,b,c^P<0.01 compared to all other treatments (mercury); ^d,e,f,g^P<0.01 compared to all other treatments (prolactin) (ANOVA with Tukey's post-test). CN: control group; MeHg: methylmercury; PRL 25 and PRL 250: 25 and 250 μg/kg prolactin, respectively, every 12 h.

Blood samples were analyzed to determine the levels of AST, ALT, urea, and creatinine (Figures 5 and 6 and Supplementary Tables S1 and S2), in addition to serum levels of mercury and prolactin (Figure 7 and Supplementary Tables S1 and S2). Serum levels of hepatic and renal markers were significantly higher in the animals of the MeHg group than in the MeHg+PRL groups, even though the levels in these groups remained higher than those recorded in the control group, regardless of sex.

A significant increase in mercury levels was observed in all the animals that received MeHg (MeHg group and MeHg+PRL groups) compared with both the control and PRL groups. There was also a significant reduction in serum levels of mercury in the MeHg+PRL groups compared to the MeHg group.

Serum levels of prolactin in control animals differed significantly from all other groups. There was a significant reduction in prolactin levels in the MeHg group. However, the serum levels of prolactin in the prolactin groups were significantly higher than those observed in the MeHg+PRL groups at the same concentrations (i.e., PRL 25 *vs* MeHg+PRL 25, prolactin 250 *vs* MeHg+PRL 250). In addition, serum prolactin levels were higher in females compared to males in all groups.

**Figure 8:**
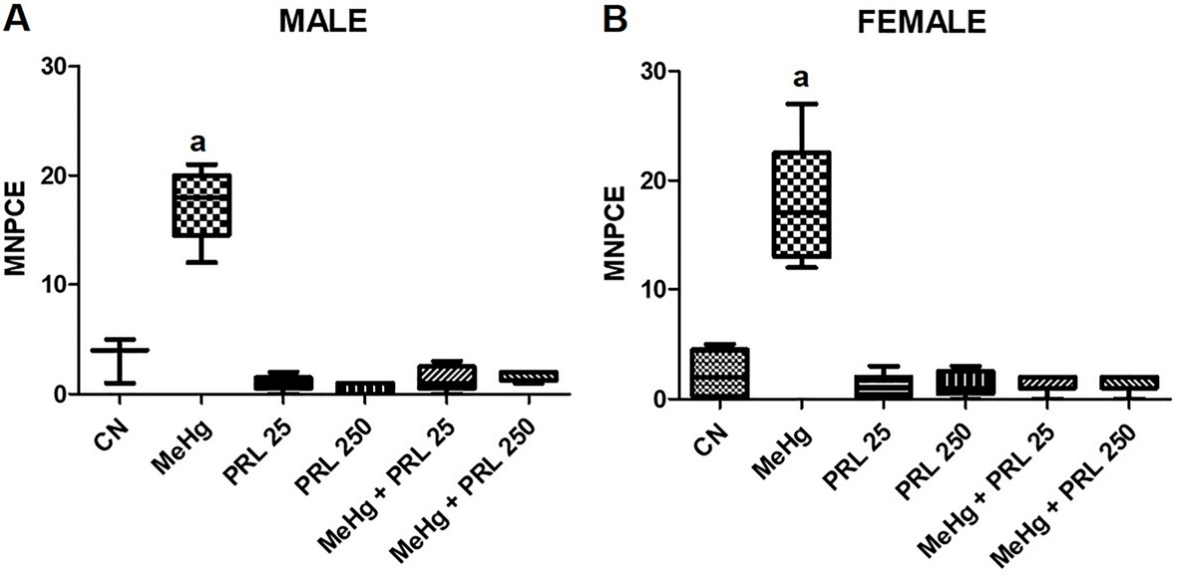
Frequency of micronucleated polychromatic erythrocytes (MNPCE) in each treatment group for males (**A**) and females (**B**). Data are reported as medians and interquartile range. ^a^P<0.01 compared to all other treatments (ANOVA with Tukey's post-test). CN: control group; MeHg: methylmercury; PRL 25 and PRL 250: 25 and 250 μg/kg prolactin, respectively, every 12 h.

The micronucleus test revealed a significant increase in the frequency of micronucleated polychromatic erythrocytes in the MeHg group compared with the control group, reflecting the mutagenic potential of this metal. In the MeHg+PRL groups, however, the frequency of micronuclei was reduced to the same levels of the control in both sexes, and at both concentrations of the hormone (Figure 8). Finally, there was no significant reduction in the percentage of PCE between treatment groups and the negative control group. It is inferred, therefore, that the cytotoxic action of the tested substances was weak or absent.

## Discussion

Prolactin initially attracted relatively little interest but increased when it was implicated along with ovarian steroids and chemical carcinogens in rodent breast cancer. Interest declined when its suppression failed to counteract breast cancer. In fact, prolactin may not cause breast cancer and may have preventive or therapeutic effects in some conditions (22). The mercury present in the biotic and abiotic environment not only compromises the survival and physiology of organisms, but also induces genetic changes. In the present study, histopathological and biochemical analyses and micronucleus test were used to evaluate the protection that prolactin exerts against the harmful effects of MeHg on the kidneys, liver, and blood of exposed mice.

### Histopathological analysis

Histopathological analysis demonstrated varied changes in renal and hepatic tissue in all treatments, although this damage was generally most frequent and intense in the animals of the MeHg group. Renal alterations promoted by MeHg, such as hypertrophied glomeruli and tubular lesions, had already been demonstrated in small rodents by Garcia et al. (23) and Khan et al. (24), respectively. Damage caused by mercury compounds, such as those found in the present study, was also identified in the liver of these mammals, (25,26). Few studies have evaluated the effects of prolactin on kidney or liver histology, given that most research has evaluated the effects of this hormone using biochemical parameters, some of which will be included in this discussion.

The tissue-level effects caused by mercury can be minimized by the action of protective agents, as observed by Al-Attar (26), which investigated the protective effect of vitamin E supplementation on mice exposed to a mixture of heavy metals (lead, mercury, cadmium, and copper) in drinking water. The results suggest that vitamin E protects against heavy metal-induced liver injuries, and the attenuating effect of vitamin E may be due to its antioxidant activity. Under the conditions of the present study, the results on the protective action of prolactin against kidney and liver damage promoted by MeHg were not conclusive and more studies should be performed.

### Biochemical analyses

As expected, the study demonstrated changes in the levels of renal (urea and creatinine) and hepatic (ALT and AST) markers in response to mercury exposure, with a significant increase in all biochemical parameters in animals exposed to MeHg. Paula et al. (27) also observed alterations in the hepatic metabolism of Wistar rats provoked by MeHg, characterized by a significant increase in the levels of ALT in the liver and blood of the animals exposed to the metal, although AST levels remained unchanged. Peixoto and Pereira (28) used hepatic and renal markers to evaluate the effects of exposure to inorganic mercury in neonatal Wistar rats and found a number of increased markers, except for ALT levels, which were reduced, in exposed rats. Our findings contrasted with this reduction in ALT level and may be related to the type of mercury used in the experiments (HgCl_2_), the exposure time, and concentration used, with a more acute exposure in the previous study, in contrast to the subchronic exposure and lower concentration in the present study.

Liver and kidney markers also often appear elevated in humans exposed to mercury (29,30). Lee et al. (29), in Korea, demonstrated a significant association between exposure to mercury and the occurrence of hyperlipidemia and high levels of ALT and AST. Li et al. (30) evaluated the renal effects of human exposure to inorganic mercury in the mercury mining area in Wanshan, China. A significant positive correlation was observed between the paired results for mercury concentrations and serum creatinine, although there was no correlation with urea.

Our results showed that the elevated levels of renal and hepatic markers observed in animals exposed to MeHg were also significantly reduced in mice treated with prolactin in a dose-dependent manner. To the best of our knowledge, the present study is the first to use liver and kidney markers to demonstrate the protective action of prolactin against mercury toxicity, but some previous studies have already shown a reduction in aminotransferases with prolactin (31,32).

Szulc-Musioł et al. (31) and Dolińska et al. (32) evaluated the addition of prolactin to the preservation solution used to wash and store rabbit and pig livers as donor organs, respectively. They concluded that hepatic ischemia and hypoxia compromise the permeability of injured hepatocytes, leading to increased levels of markers such as ALT and AST in the blood or in the preservation solution. In both studies, the prolactin added to the preservation solution resulted in a significant reduction in transaminases present in the fluid, reflecting the reduction in the release of these enzymes by the organ and, consequently, a delay in the degeneration of hepatocytes.

Another type of approach was used by Yang et al. (33), who evaluated the relationship of prolactin with various metabolic parameters in women with polycystic ovary syndrome. When performing Spearman's correlation analysis, the authors found a significant negative correlation between the prolactin levels and ALT and AST levels.

It was clear in the present study that there was a reduction in biochemical parameters (ALT, AST, urea, and creatinine) in the groups of animals exposed to the combined treatment compared to the MeHg group. Furthermore, the higher the dose of prolactin, the greater the reduction in the level of these parameters, approaching the levels observed in the control group and demonstrating the protective effect of prolactin against MeHg toxicity in the liver and kidneys of exposed mice. It is evident, therefore, that the damage in liver and kidney tissues (as mentioned above) in the PRL and MeHg+PRL groups was not sufficient to significantly alter the functioning of the kidneys or liver of these animals, as observed in the MeHg group.

Serum mercury level is an important indicator of exposure to organic mercury (34) and several *in vivo* experimental studies have demonstrated a significant increase in mercury levels in the blood of exposed animals (27,35). This situation was confirmed in the present study, since serum mercury was significantly higher in the animals of the MeHg group than in all the other groups, with no differences between males and females. Mercury also accumulated in other tissues and organs and can vary greatly. Paula et al. (27) evaluated the accumulation of mercury in the blood, kidneys, and brain of rats exposed to MeHg, finding higher levels in the brain. In the study by Barcelos et al. (35), also with rats exposed to MeHg, blood and liver were analyzed, and higher levels were found in the blood.

Serum prolactin levels were significantly higher in the PRL groups compared to the control, although these levels showed some decrease in the MeHg+PRL groups. Blood prolactin levels showed no significant variation between male and female control groups but were significantly higher in females in all other experimental groups.

### Micronucleus test

The genotoxic effect of MeHg exposure was reflected in the increased number of MNPCE in exposed mice. Many studies have already demonstrated the genotoxic effects of mercury in fish (5,6), rats *in vivo* (35) and *in vitro* (36), humans (37), and tadpoles (38). The genotoxicity of mercury was also demonstrated by the comet assay (6,35,36). Chromosomal aberrations and polyploidy have also been considered characteristic parameters of the genotoxicity of this metal (2,13). These studies indicate that the genotoxic effect may be related to the production of reactive oxygen species that, in turn, induce DNA breaks, in addition to the adverse effect on tubulin, the structural subunit of microtubules involved in cytoskeleton organization and cell division.

Previous studies have shown that prolactin may act as a co-mitogenic factor, favoring cell proliferation (11-13). Bitgen et al. (39) also reported that, by intensifying cell proliferation, high levels of prolactin could increase the number of DNA replication errors and cause aneuploidy, being responsible for the higher frequency of micronuclei. However, the present study demonstrated a clear protective effect of prolactin against the genotoxic impact induced by MeHg, significantly reducing the number of MNPCE in the bone marrow of mice (of both sexes) in the MeHg+PRL groups. Similar results were presented by Silva-Pereira et al. (13), who observed the reduction of genotoxic effects *in vitro* induced by MeHg in the HL-60 human leukemia cell line and in normal human lymphocytes after treatment with prolactin. This protective effect may be related to the activation of pathways through the interaction of prolactin with its receptor, as in the JAK-Stat and MAPK pathways, which are involved in the transcription of cyclins, in the activation of guanine nucleotides, and in the enzymes that detoxify and organize the cytoskeleton, which may then restrict DNA damage (directly or indirectly) and apoptosis (13,40).

Overall, the evidence from the histological, biochemical, and genotoxic parameters analyzed in the present study indicated that prolactin had protective properties against the toxic effects of MeHg. However, further research is necessary to better determine the effects of this hormone. The contradictory relationship between prolactin and breast cancer, for example, deserves consideration in case of a future proposal for prophylactic use of the hormone. It is worth remembering what happened with the ovarian steroid. Goodman and Bercovich (22) warn that long-known estrogen-related cancers of the ovaries and breast have not prevented the widespread use of estrogen for contraception and supplementation.

Therefore, even considering the possible side effects of high levels of prolactin suggested in previous works and that the results of our histopathological analyses were not enlightening, the excellent results obtained in biochemical and genotoxic analyses of the present study allowed us to suggest the continuity of the research for a future use of prolactin as an alternative to prevent the damage caused by mercury, especially in populations that are more exposed.

In our future studies of this nature, oxidative stress markers for enzyme bioassays will be included and the expression of the Ras/Raf/MEK/MAPK kinase pathway will also be studied to better characterize the protective effect of prolactin, including in other tissues, evaluating, among others, the antiapoptotic and neuroprotective actions.

## Supplementary Material

Click here to view [pdf].
